# Editorial: Advancing non-small cell lung cancer management through biomarker integration

**DOI:** 10.3389/fonc.2026.2005040

**Published:** 2026-10-15

**Authors:** Prashanth Ashok Kumar, Sora Ely, Alina Basnet

**Affiliations:** 1 George Washington University Hospital, Washington, DC, United States; 2 SUNY Upstate Medical University Hospital, Syracuse, United States; 3 Guthrie Cortland Medical Center, Cortland, United States

**Keywords:** artificial intelligence (AI), biomarker, molecular alterations, non small cell lung cancer (NSCLC), radiomics

Non-Small cell lung cancer (NSCLC) has been in the forefront of biomarker research with a dozen plus biomarkers based on which, a targeted therapy is determined (1). This encompasses all stages of NSCLC from early resectable adenocarcinoma to widely metastatic disease( (2), Li et al). Tools like transcriptomic profiling has created rsik groups even within a particular disease stage (Li et al). In disease that can be surgical resected, Epidermal growth factor receptor (EGFR), Anaplastic Lymphoma Kinase (ALK), have impacted management in both the adjuvant and neoadjuvant space with Osimertinib and Alectinib respectively (3). In 2026, Selpercatinib demonstrated meaningful benefit when used adjuvantly in Rearranged During Transfection (RET) fusion positive NSCLC (4). In the metastatic setting, the National Comprehensive Cancer Network (NCCN) guidelines list 13 molecular markers beside PDL–1, HER2 and MET Immunohistochemistry (IHC) based on which treatment decisions are made (5). Even within a particular mutation group like EGFR, there can be variations like EGFR exon 20 insertions that has poor outcomes with suboptimal response to exiting targeted agents (Campos Balea et al.). Beside the guideline recommended biomarkers, treatment decisions in real life situations also factor in novel markers with established translation science (6, 7). These include but not limited to markers like combinations of Tumor Mutational Burden and PD–L1 (8), Tumor Infiltrating Lymphocytes (TILs) (Gauthier et al.), STK11, KEAP1 and other markers that predict immune–checkpoint therapy (ICI) resistance (7), and markers predictive of tumor microenvironment include artificial intelligence (AI) tools (9). Biomarkers that help understand tumor biology is also pivotal. CCDC58 for instance, when upregulated resulted in cell proliferation (Dai et al.). Indoleamine 2,3–dioxygenase–1 expression in both tumor and immune cells may have a prognostic role correlating with survival (Agustoni et al.). F2, GPX3, KMO, and KYNU are novel genes with a prognostic role where all 3 besides F2 are downregulated in adenocarcinomas of the lung. Their expression may impact sensitivity to chemotherapeutic agents like cisplatin and gemcitabine (Chen et al.).

Research topics and studies focusing on integrating novel biomarker strategies into the prognostic and predictive algorithms may help the field by redirecting research interest(Zhou and Jiang, (10)). Traditional markers like CEA may have limited utility in the modern era of NSCLC management despite some analysis demonstrating that increasing levels correlate with worse outcomes (Ma et al.). Xi ZH et al. argues that biomarkers that help appreciate the biologics of tumor microenvironment must be integrated into staging, prognostication, and eventually treatment decisions (Xi et al.). Resistance to treatments and disease recurrence is orchestrated by an interplay of multiple pathologic, immunologic, and molecular characteristics that may be inherent to the tumor or may develop overtime. A simple anatomic staging based on the tumor size, lymph node status, or distant spread alone may not capture this complexity (Xi et al.). While the importance of tumor microenvironment is increasingly being recognized, the review suggests that equal importance should be given to the lymph node immune microenvironment and highlights that germinal center lymphocyte processing of tumor antigens in the lymph nodes may play an important role in lung cancer progression. In fact, lymphocyte microenvironment may provide valuable information, even if the lymph node is not involved with cancer(Xi et al.). This hypothesis is supported by other studies that have shown that lymph nodes that drain the tumor have an immunosuppressive environment, where there are significantly higher number of regulatory diesel’s compared to the cytotoxic ones. Potentially contribute to ICI resistance (11). Several biomarkers that detect the various arms of the immune response are described like VEGF, MMP9, PDL1, CD11, C66, etc are available. But their utility and practical use is uncertain. A standalone marker is usually limited in its ability to a discern a complex interplay of various biologic factors. 21^st^ century technologies and AI powered tools could potentially simplify this complexity. Single–cell and multimodal transcriptomics, Sequencing–based spatial transcriptomics and Imaging–based/high–plex proteomics are examples of such tools that may help refine tumor classification beyond histology, IHCs and individual mutations (Xi et al.). These technologies are analogous to google earth as they map out and magnify the detailed molecular and immunologic activity down to a very minute level in every physical coordinate of a tissue sample (12). Multi–omics data can define molecular subtypes of NSCLC and can identify potential novel targets like domain–negative anillin protein (dnANLN), the expression of which has shown susceptibility to docetaxel *in vitro*(Ma et al.). Technological limitations remain. But clinical, biologic and biotechnological research must utilize these tools moving forward with an eventual converging aim to improve patient outcomes (Xi et al.).

Radiomics is a tool that converts clinical images such and CT and PET images into high fidelity quantifiable data, enabling correlation with molecular markers, histopathologic correlates and other clinically relevant parameters( (13), Zollinger et al.). Through this tool, clinical images can be used as a surrogate for markers such as EGFR, TMB, cancer invasiveness, distinguishing benign vs aggressive lesions, occult metastasis, etc(Zollinger et al.). Radiomics tools such Computer–Aided Nodule Assessment and Risk Yield (CANARY) has utilized images to predict mutations in TP53, KRAS and EGFR (14). In clinical practice, oncologists frequently run into scenarios where sufficient tissue, tumor cell and DNA are not available to perform comprehensive sequencing and all the necessary IHCs listed in the guidelines for NSCLC (15). Blood based circulating tumor DNA (DNA) is not always sensitive, particularly for fusions where RNA based analysis on tissue is needed (16). Patients may not always be willing to undergo a repeat tissue biopsy for the sake of sequencing (15). Radiomics may have a role in such niche clinical situations (Zollinger et al.). A recent study demonstrated that radiomics could identify NSCLC patients at risk for recurrence after neoadjuvant chemotherapy with ICI (17). Radiomics thus can be therefore serve as a non–invasive, cost–efficient adjunct tool in prognostication of NSCLC(Zollinger et al.).

In summary, improving outcomes in NSCLC is closely tied to advances made in biomarker recognition and targeting. Future studies should embrace modern technology and provide clarity to clinicians on how to utilize them in clinical practice (18).
